# Exogenous Factors Affecting the Functional Integrity of Male Reproduction

**DOI:** 10.3390/life11030213

**Published:** 2021-03-09

**Authors:** Filip Tirpák, Hana Greifová, Norbert Lukáč, Robert Stawarz, Peter Massányi

**Affiliations:** 1 AgroBioTech Research Centre, Slovak University of Agriculture in Nitra, Tr. A. Hlinku 2, 949 76 Nitra, Slovakia; 2 Department of Animal Physiology, Slovak University of Agriculture in Nitra, Tr. A. Hlinku 2, 949 76 Nitra, Slovakia; hana.greifova@uniag.sk (H.G.); norbert.lukac@uniag.sk (N.L.); 3 Institute of Biology, Pedagogical University of Krakow, Podchorazych 2, 30-084 Krakow, Poland; robert.stawarz@up.krakow.pl

**Keywords:** heavy metals, radiation, endocrine disruptors, mycotoxins, testes, spermatozoa, seminal plasma, oxidative stress, mode of action, risk factors

## Abstract

Natural processes along with increased industrial production and the irresponsible behavior of mankind have resulted in environmental pollution. Environmental pollutants can be categorized based on their characteristics and appearance into the following groups: physical, biological, and chemical. Every single one of them represents a serious threat to the male reproductive tract despite the different modes of action. Male gonads and gametes are especially vulnerable to the effect of exogenous factors; therefore, they are considered a reliable indicator of environmental pollution. The impact of xenobiotics or radiation leads to an irreversible impairment of fertility displayed by histological changes, modulated androgen production, or compromised spermatozoa (or germ cells) quality. The present article reviews the exogenous threats, male reproductive system, the mode of action, and overall impact on the reproductive health of humans and animals.

## 1. Introduction

The present society is largely focused on the creation and optimization of technological methods. This effort for financial or personalistic gain, or the process simplification of production brings along high, although on first sight, imperceptible sacrifice. The development and spread of diseases of affluence reflect the negative impact of various byproducts of industrial production or other anthropogenic activities. Accordingly, the most advanced industrial areas are usually also known for the enormous environmental pollution. The accumulation and consecutive synergism of toxicants in the living environment leads to impairment of individual physiological processes or even death [1].

Contaminants, commonly known also as risk factors, may have different characteristics depending on their origin. Physical contamination is caused by ubiquitous ionizing and nonionizing radiation. Micro-organisms and their metabolites or pollens can be considered as risk factors of biological origin. Chemical contaminants include endocrine disruptors or toxic metals [2].

Pollutants tend to accumulate in different organs and harm their functions. Poisoning or contaminant-derived diseases can be acute (episodic) or can develop over time (chronic intoxication). They are often accompanied by skin problems, breathing complications, convulsions, digestive disorders, or serious failures of the central nervous system and endocrine activity. The most terrifying effects, affecting even the progeny, are mutagenicity and carcinogenicity [3,4,5,6,7,8].

A good example of the consequence of high environmental pollution is the year to year decrease in the infertility of wild animals, domestic animals, or even humans. A negative effect of toxicants (decrease in concentration, motility, or longevity of spermatozoa) is often monitored on the individual or the group of individuals, however, the mechanism of action of toxicants on the cellular level is not studied enough. If so, most of the time only one compound is studied at a time and studies do not take into consideration the possible effect of synergism or antagonism of xenobiotics [9,10,11,12].

Previous studies imply that the exogenic effect of toxicants is strongly related to the enhanced production of reactive oxygen species (ROS) and reactive nitrogen species (RNS), resulting in the degradation of biomolecules or even apoptosis [13]. On the other hand, it is important to mention that ROS are necessary for the physiological processes of spermatozoa such as capacitation or acrosome reaction. Therefore, spermatozoa may be affected by several exogenic and endogenic factors where the determining variables are the imbalance between antioxidants and free radicals and appropriate proportion between individual components of seminal plasma [14].

The present article describes exogenous agents in general and summarizes the current knowledge about their effects on male reproductive system and their modes of action.

## 2. Environmental Pollution

The pollution of the environment has a serious consequence in the endangerment of human and animal health. It is caused primarily by growing industrial production, transportation, chemical substances used in agriculture [15,16], and pharmaceutical remedies [17]. Soil contamination and thus the pollution of foods of plant origin is one of the most pressing issues in discussion about food safety on the European [18] or even global [19] level.

Gonads and gametes serve as a very sensible and reliable barometer of the incidence of risk elements in the environment. They are affected via the degeneration of seminiferous epithelium, abortion of connection with the basal membrane, defects in spermatozoa development, and generation of ROS thereby reducing male fertility [20,21,22,23]. Donkin and Barres [24] report that environmental factors along with diet and lifestyle are reflected in spermatozoa epigenetics. More important, these genetic changes may be passed on to the next generations via epigenetic inheritance. We recognize three types of contaminants that can eventually cause environmental pollution and we divide them based on their character and appearance on physical biological and chemical contaminants [2].

### 2.1. Physical Contamination

The Glossary of Environment Statistics issued by the United Nations Department for Economic and Social Information and Policy Analysis defines physical pollution as pollution caused by color, suspended solids, foaming, thermal conditions, or radioactivity [25]. This definition was adopted by the Organization for Economic Cooperation and Development (OECD) and is still used today [26]. Color pollution is the result of color contamination by an inappropriate arrangement of colors that induces a disorder in the perception of the visual field within the natural or urban environment [27]. Suspended solids are contaminants of the water environment in the form of microplastics. Microplastics are serious polluting agents themselves, moreover, they may have a role as pathogen carriers. Their presence in a marine environment is a threat not only to aquatic flora and fauna but also to human food safety in the form of seafood and salt [28,29].

Temperature changes in the aquatic system may also cause thermal pollution which is reflected in degraded water quality. The source of thermal pollution is frequently found in the activity of power plants. The negative effect is then shown on soil erosion or on fish that exhibit thermal shock, metabolism alterations, and reproductive dysfunction [30,31]. Another water-related contaminant causing physical pollution is foaming. Due to the appearance of the foam on the surface of the freshwater, contamination can be easily visually recognizable. Foam pollution may find a cause in the natural processes of aquaculture or anthropogenic activity. Man-made pollution is often caused unintentionally by of oil, detergent, or lignosulfonate leaks. Foam lines are formed from the surfactants which reduce the tension of the water surface and enable the foam bubbles occurrence [32].

Radiation is an extensively occurring contaminant of the environment. Radiation can be divided based on the amount of emitted energy into ionizing and non-ionizing, which determinates the power and further features of the radiation. Non-ionizing radiation causes electron excitation which can induce heat generation. Ionizing radiation possesses enough energy to induce the emission of electrons from atoms and electrons being a threat to all living organisms [33,34]. Radioactive compounds are usually the waste of nuclear power generation, production of nuclear fuels, weapons development, biomedical interests, and industrial activities. However, radioactive contaminants can also originate in nature [35]. Radiation is the only exogenous agent of the group of physical contaminants that have a direct effect on male reproduction. As the ubiquitous aspect of the modern age, radiation may be considered a serious threat based on its emitted energy, frequency, and dose of exposure (Table 1).

### 2.2. Biological Contamination

The term biological contaminant covers all cases of contaminations induced by the biological activity of organisms in the environment. This includes all invasive plant and animal species, pollen, but concerning reproduction, the most frequent biological pollutants are microorganisms. Either microbes themselves or their metabolites cause air, water, soil, or food pollution [54,55,56,57]. The food chain contains numerous entries for contamination. The contamination of groceries is often associated with improper storage conditions while fresh products are considered safe and healthy. This bias is frequently promoted along with a healthy lifestyle. However, microbiological threats are present in every step of the farm-to-fork chain, including the soil, water sources, cultivation, harvesting, and processing [58]. In respect to one of the highest sources of contamination, water, the US Environmental Protection Agency identifies over 500 waterborne pathogens, including viruses, fungi, bacteria, protozoa [59]. Epidemiologists also warn against the HVAC (heating, ventilation, and air conditioning) technology and hand dryers in public restrooms when they are not regularly maintained, inspected for microbial pollution, and disinfected [60].

Male reproductive organs are prone to microbial infection and subsequential inflammations. The male urogenital tract is occupied by numerous bacteria including *Escherichia coli*, *Proteus* spp., *Enterococcus* spp., and other Gram-positive and Gram-negative bacteria. The presence of bacteria in the semen is called bacteriospermia and does not inevitably mean the pathological sign. On the contrary, the presence of *Mycoplasma* spp., *Chlamydia* spp. or other pathogenic bacteria are not appreciated. Sequela such as orchitis, epididymitis may be caused by *Brucella* spp., *Mycobacterium leproe*, *Mycobacterium leproe*, or uropathogenic *Escherichia coli*. In addition to epididymis and testis, *Chlamydia trachomotis* targets and causes inflammation of the urethra, prostate, and seminal vesicles. In certain cases, when the concentration of pathogenic bacteria is too excessive, the body responds with leukocytospermia—abnormally high concentration of white blood cells in ejaculate. The physiological regulation of this phenomenon is not sufficiently explained yet [60,61,62,63]. Some authors even did not find any or just weak associations between bacteriospermia and leukocytospermia [64,65].

Semen may be considered the vector of viral infection; however, the male reproductive system may also suffer from viral contamination. It has been documented that semen may be a host to approximately 27 viruses. This includes Adenoviruses, Ebola virus, Hepatitis virus (HPV) B and C, Zika virus, Epstein Barr virus, Human immunodeficiency virus (HIV), Mumps virus, several herpes viruses, Human T-cell lymphoma virus, SARS-CoV-2 virus, etc. [61,66,67]. Evidence on the direct effect of viruses on fertility has been reported by several studies. Some viruses affect only the spermatozoa by alteration of their motility and viability (Hepatitis B and C, SARS-CoV-2, HPV) [67,68,69]. On the other hand, the CoxsacKie virus, Mumps virus, HIV, Zika virus, HPV, Influenza virus target male reproductive organs—testis and epididymis [68,69,70,71,72]. In some cases of HPV infection, the targeted site might be just Sertoli cells. This phenomenon is called the “Sertoli cell-only” syndrome [71].

### 2.3. Chemical Contamination

Chemical contaminants turn into pollutants when accumulations are sufficient to undesirably affect the natural environment or to present a risk to living organisms. There are thousands of industrial chemicals identified as a hazard to humans, animals, and the environment. Therefore, governmental agencies regulate their production, storage, transportation, and disposal. Sources of chemical contamination contain agricultural activities, industrial and manufacturing activities, municipal waste, service-related activities, and resource extraction [73].

#### 2.3.1. Heavy Metals

The term “heavy metals” defines a wide group of contaminating elements that are distinctive of their miscellaneous features, associated effects, and origin. Heavy metals of-ten appear as positively charged molecules that bind to negatively charged molecules of other elements to become a part of a compound. Ecotoxicological studies include the group of heavy metal elements like Cu, Zn, Cd, Hg, Pb, Cr, Ni, Mn, Fe, and semimetals As and Se. These metals are sometimes incorrectly indexed as toxic metals which is inaccurate since many metals are essential nutrients and at certain levels are not toxic at all [74,75]. Heavy metals fall into a group of trace elements. Their impact on the body may be diverse—metabolic, carcinogenic, or even mutagenic. The mode of action depends on several factors: the features of the element, a form of contact with the organism, the dose of the element, exposure time, type of absorption of the element, or bioaccumulation. The current research is aimed mainly at heavy metals present in industrial or former mining areas [76]. According to Arvay et al. [77], toxicity varies in dependence on mobility, solubility, pH, etc. Numerous studies proved that heavy metals accumulate in adipose tissues and consequently disrupt the functioning of internal organs and impair the nervous or endocrine system [78,79,80]. In general, compounds containing heavy metals are toxic, mutagenic, teratogenic, and carcinogenic for animals [81]. There are several ways of the entrance to the animal organism (e.g., consumption, inhalation, through the skin) that can cause severe intoxications [82]. Therefore, thorough, and periodic monitoring of health conditions is required in polluted areas [9,16,83]. The detrimental effects of heavy metals have been reported by numerous studies. Table 2 displays the most serious heavy metals-induced deteriorations reported in association with the male reproductive system.

#### 2.3.2. Endocrine Disruptors (EDs)

Endocrine disruptors represent a large group of environmental xenobiotic com-pounds that can interfere with the biosynthesis, secretion, action, or metabolism of endogenous hormones, which ultimately leads to changes in the efficacy of these hormones [120,121]. EDs can be of natural origin (phytoestrogens, hormones, etc.) or are the result of anthropogenic activities (pharmaceuticals, dioxins, pesticides, etc.) [35]. Because most of them can interact with cell surfaces or nuclear receptors, they can cause alterations in cellular homeostasis even at extremely low concentrations, which places them in a special category of toxic substances in terms of health risks [122]. These substances are characterized by persistence and bioaccumulation properties, which are associated with the inhibition and disruption of physiological processes at the level of cells and molecular signaling pathways. The results are irreversible changes in the endocrine, cardiovascular, nervous, reproductive, and immune systems [123]. Due to their ability to interfere with estrogen, progesterone, and androgen receptors, endocrine disruptors pose extremely serious health risks in association with impairment of male reproductive functions [124]; at the forefront of discussions is infertility due to reduced sperm count, reduced sperm quality, and inhibition of steroidogenesis [7,125,126]. Many xenobiotics have endocrine disrupting properties, including industrial chemicals, solvents, polycyclic aromatic hydrocarbons, plasticizers, and compounds found in detergents, cosmetics, agricultural chemicals, and pesticides, as well as natural and synthetic hormones and drugs. These chemicals are widely distributed in the environment, especially through the trophic chain and wastewater [127,128]. At present, the attention of science in the field of EDs is focused mainly on synthetic industrial chemicals because of the absence of evolutionary adaptation to these xenobiotics [129]. These are plasticizers and other chemicals present in plastic as well as paper food packaging, which expose the human population to endocrine disruptors through food practically continuously [130]. This phenomenon has been confirmed by numerous studies, the results of which indicate increased concentrations of bisphenols, alkylphenols, phthalates, and perfluoroalkyl compounds in the saliva, urine, and milk of exposed individuals, but also in the food of daily consumption [131]. EDs also include many agrochemicals and pesticides, which have been shown endocrine disruptive activities in the living organism [132]. In particular, first-generation pesticides are a dangerous heritage for today’s population because they persist in soils, aquatic sediments, bioaccumulate in invertebrate and vertebrate tissues, and move up in trophic chains [133,134]. Numerous studies confirm that the increasing number of compounds released uncontrollably into the environment every year, which we classify as endocrine disruptors, harm animal and human health, and it is necessary to monitor their effects and consequences in organisms [7,135,136,137].

## 3. Mode of Action (MoA)

As listed earlier, there are three types of contaminants (physical, biological, and chemical) based on their characteristics, origin, and MoA. This implies their various effects on the functionality of organs and gametes. The biological system has its own mechanisms to prevent the toxicity of the reproductive system. The hematotesticular barrier (HTB) regulates the migration of some toxicants from blood to the testis, mature sperm chromatin is inactive and firmly coiled, defective spermatocytes are degraded and replaced. The imperfection of the HTB consists in the influx of lipophilic compounds allowing the exposure of vulnerable spermatogonia to toxicants and compromises male fertility [74]. HTB is one of the tightest barriers between blood and tissue, dividing seminiferous epithelium into the basal and apical parts. Also known as the blood–testis barrier, HTB differs from other tissue barriers by its structure, composed of tight junctions, ectoplasmic specializations, desmosomes, and gap junctions. This highly specific and unique structure provides a favorable microenvironment for meiosis and following the development of spermatids into spermatozoa [138]. Spermatogonia are localized in the basal compartment while primary and secondary spermatocytes along with round and elongating and already elongated spermatids are situated in the apical compartment [139].

### 3.1. Physical Pollutants

The MoA of radiation on male reproductive physiology is not clearly stated across the scientific literature. While ionizing radiation is considered dangerous due to the ability of electron liberation, non-ionizing radiation is supposedly safe as it lacks photon energy. However, non-ionizing radiation excites electrons which generates heat. It is assumed that heat generation stimulates OS production [33,44,140]. The spermatozoa of radiated individuals may contain microtubules with the modified arrangement, furthermore, disrupted mitochondria with up-regulated ROS generation may appear in radiation-exposed spermatozoa [141]. Excessive OS initiates adverse actions towards testicular tissue, mainly Sertoli cells, Leydig cells, and germ cells. The modulated integrity of testis indicates impaired spermatogenesis, apoptosis, and production of spermatozoa with functional and morphological damage. Moreover, male gametes may possess defected DNA [36,37,48,52]. A decreased number of viable Leydig cells causes alteration of testosterone production. This variation in androgen production impairs the whole hypothalamic-pituitary-adrenal axis [142]. The significant limitation of the topic of radiation and male reproduction arises in the scientific community. Biologists evaluate the risk of radiation based solely on the biological data (mainly OS production) while physicists look at the same problematics on the level of electrons with no biological relevance.

### 3.2. Biological Pollutants

Biological pollutants affect reproduction primarily from the microbial perspective. Bacterial contamination of the urogenital tract is associated with an enhanced level of leukocytes in the semen. Leukocytes generate ROS in their combat with an infection which may cause deleterious changes of spermatozoa and the integrity of their DNA [64]. The effect of mycotoxins on male reproduction needs to be remembered as well. As shown by Zheng et al. [143], zearalenone impairs reproductive functions. The results of in vitro administration of zearalenone were demonstrated on the Sertoli cells. Irreversible damage was detected damage to the cytoskeletal structure, namely the absence of both mitochondria and Golgi apparatus, and relocalization of vacuoles into the cytoplasm. Moreover, mycotoxins can interfere with the secretory functions of Sertoli cells. Most viruses affect only spermatozoa if at all. However, few other types target testicular and epididymal tis-sue and induce severe inflammation [68,69]. This process includes HTB, which forms an interface between the immune system and germ cells. Strong inflammation in combination with responding cytokines modulates the functionality of HTB. Comprised barrier enables the entrance of inflammation and may result in the formation of granulomas as a response to chronic inflammation. Moreover, inflammation induces excessive ROS pro-duction causing even superior damage to the testis, germ cells, and developing spermatozoa [144].

### 3.3. Chemical Pollutants

Chemical pollutants are probably the most recognized of all pollutants. Toxic heavy metals, due to their capability to accumulate in tissues, represent a threat as chronic toxicants. The toxic mechanisms of heavy metals are defined as ion imitation, interruption of cell signaling pathways, oxidative stress, altered gene expression, apoptosis, disruption of the testis–blood barrier, and inflammation. It has been previously revealed that toxic heavy metals alter Leydig cell development and induce Leydig cell tumors. Even low doses of these toxic agents alter the walls of seminiferous tubules, thinness the germinal epithelium, thus impairing the process of spermatogenesis. Heavy metal exposure also results in the altered weight of testes, cellular degeneration, interfered androgen hormones pro-duction, dilatated and congested blood vessels, and necrosis [9,89,101,145]. Elevated environmental concentrations of heavy metals are markedly displayed in the enhanced level of OS in seminal plasma [146].

Endocrine disruptors are associated mainly with imperceptible regulation of endocrine systems. The chemical structure of these pollutants mimics the natural hormones or interfere with the synthesis and secretion of the natural hormones. These agents are responsible not only for the improper development of sexual organs of the affected individual, but their effect may be also displayed on the health and sexual status of its progeny [7,137,147]. In addition to chemicals as polychlorinated bisphenols, organochlorine pesticides, and plasticizers and nonylphenols, it has been proposed, that heavy metals mercury, cadmium, and arsenic may also possess endocrine system modulating functions [148,149]. Several studies report the direct disrupting effect of EDs on the germ cells and Leydig cells via excessive generation of OS [150].

### 3.4. Oxidative Stress—A Common Feature

Oxygen is a biogenic element for all aerobic cells. It is necessary for the maintenance of normal cell functions, however, reactive oxygen species (ROS) which are formed as a by-product of the natural metabolism of oxygen may be harmful to the cell. The deciding factor is whether there is a balance between ROS generation and antioxidants which can scavenge free radicals [151]. Free radicals may be defined as an independently existing atom or molecule with an unpaired electron. By accepting or donating the electron molecule becomes a free radical. Reactive oxygen species and reactive nitrogen species (RNS) belong among the best known and most frequent free radicals [152]. ROS represent a wide category of molecules including radicals and non-radicals. Oxygen-derived radicals comprise hydroxyl ion (^•^OH), superoxide (O_2_^•−^), peroxyl (LOO^•^), hypochlorous acid (HOCl), etc. Non-radicals include ozone (O_3_), singlet oxygen (^1^O_2_), peroxide substances (H_2_O_2_), and others [152,153]. Nitrogen-derived radicals are nitric oxide (^•^NO), nitrous oxide (N_2_O), peroxynitrite (ONOO^-^), nitroxyl anion (NO^−^), and peroxynitrous acid (ONOOH).

Metabolism and the utilization of oxygen is an essential requirement of male gametes. Free radicals play an important role in several physiological processes of the reproductive tract [154]. Spermatozoa themselves generate ROS which is necessary for capacitation, hyperactivation, acrosome reaction, and sperm-oocyte fusion [14]. Each ejaculate contains potential sources of ROS. Often, these sources are leukocytes and neutrophils, which apply their cytotoxic mechanism against cells and pathogens and produce high levels of ROS [155].

The endogenous production of O_2_^●−^ is caused by cell respiration. This radical is by itself relatively non-reactive, but the presence of H^+^ leads to the formation of H_2_O_2_, which is largely involved in the lipid peroxidation of the plasma membrane [156,157] abundant in PUFA [158,159]. Gosalvez et al. [14] report that by the action of enzymatic antioxidants H_2_O_2_ is catalyzed to water and oxygen. The external sources of ROS are associated with an individual’s diet and the environment in which the individual lives. Indirect and direct effects on male infertility are induced by industrial intermediate products and wastes [160], smoking and alcohol consumption [161,162,163], obesity [164], diabetes [165], physical exercise [166], psychological stress [167], aging [168], or the presence of increased levels of toxic heavy metals in the environment [169,170].

NO, the major RNS, is formed of L-arginine by redox-reaction via nitric oxide synthase (NOS). This reaction necessitates oxygen and several cofactors. RNS are invaluable for numerous physiological processes in male reproduction, demonstrating their importance in intracellular signaling pathways [142]. Moreover, NO regulates the timing of the opening and closing of tight junctions of HTB thus prevents xenobiotics from entering the seminiferous tubules. Further, RNS are similarly as ROS involved in capacitation, acrosome reaction, hyperactivation, and oocyte-spermatozoa fusion. An excessive amount of RNS results in stimulation of apoptosis, inhibition of Leydig cell steroidogenesis. Clinically, RNS is often associated with leukocytospermia, varicocele, and erectile dysfunction [171].

The exposure to oxidative stress may result in adaptation (increased activity of defense mechanisms), damage to macromolecules (DNA fragmentation, protein modifications, lipid peroxidation) [172,173], or cell death (apoptosis, or even necrosis with a wider impact on surrounding cells and tissue) [174]. The effect of OS may be also mitigated by antioxidants by oral administration [175,176,177,178] or direct supplementation of reproduction-associated cells [179,180,181,182,183].

## 4. Conclusions

Environmental pollutants have been determined as a risk factor to the overall health status of both humans and animals. The synergism of several contaminants and their bioaccumulation may lead to the induction of oxidative stress, resulting in cellular disruption, histological damage, endocrine disruption, and possible transgenerational effects. It is necessary to also consider antagonistic effects. The reproductive system, due to its high sensitivity, is considered the plausible marker of environmental quality that is applicable for humans as well as for various domestic and wild animals. Plenty of xenobiotics target the testis and impair their function. The male reproductive system possesses molecular repairing mechanisms that enable the limited but continuous production of viable gametes. Nevertheless, it is important to monitor and protect the environment by all means in order to maintain the fertility of humans and animals.

## Figures and Tables

**Table 1:** The effect of non-ionizing and ionizing radiation on the most targeted sites in male reproduction.

Radiation	The Site of the Effect		
	Testis	Epididymis	Spermatozoa
Non-ionizing	dilatated and congested blood vessels in tunica albuginea and interstitium degenerated spermatogenic cells diminished Sertoli cells containing numerous vacuoles, swollen mitochondria, and broken organelles enhanced production of cytokines by Sertoli cells germ cells arrested in pre-meiotic stages induced generation of ROS [36,37,38]	reduced weight of epididymis decreased sperm count morphological defects of spermatozoa increased lipid peroxidation diminished content of glutathione degeneration of epithelium cells [39,40,41,42,43]	dose & frequency-dependent effect on motility spontaneous acrosome reaction sperm head malformations increased DNA damage [33,36,44,45,46]
Ionizing	decreased testis weight damaged seminiferous tubules disorganized spermatogenic cells declined number of spermatocytes and spermatogonia induced apoptosis of spermatogenic cells degeneration of Sertoli cells the high appearance of swelling mitochondria extensive ROS generation [47,48,49,50]	the lower weight of epididymis diminished luminal diameter high incidence of vacuoles in the epithelium impaired spermatogenesis reduced sperm count elevated apoptosis enhanced intracellular ROS decreased level of zinc [47,51,52,53]	decreased motility and viability reduced sperm count morphological malformations elevated intracellular ROS down-regulated expression of tubulin decreased content of ATP [47,49,50,51]

**Table 2:** The effect of selected (the most harmful) heavy metals on the most targeted sites in male reproduction.

Heavy Metal	The Site of the Effect		
	Testis	Epididymis	Spermatozoa
Cadmium	alterations in the number and structure of seminiferous tubules the increased volume of stroma the thinned layer of Sertoli and germ cells altered spermatogenesis [84,85,86]	enhanced weight of epididymis thickened epithelia widened interstitium vacuolized Sertoli cells impaired spermatogenesis dilatated blood vessels induced apoptosis and inflammation interfered androgen production increased ROS production [87,88,89,90]	reduction in sperm count decreased viability and motility altered morphology altered acrosome and mitochondrial segment changed DNA methylation [91,92,93,94,95,96]
Lead	reduced testis weight disrupted testosterone production elevated ROS disorganization of seminiferous tubules interfered or even absent spermatogenesis enlarged spermatocytes inflamed tunica albuginea down-regulation of Catsper 1 and 2 [97,98,99,100,101,102]	decreased weight of epididymis increased oxidative stress and lipid peroxidation diminished epididymal spermatozoa modulated enzymatic activity reduced quantity of stereocilia of epithelial cells lowered number of spermatozoa [103,104,105]	decreased sperm count and motility excessive generation of ROS enhanced DNA damage morphological malformations decreased intracellular cAMP inhibition of sperm creatine kinase displacing zinc from its binding sites [101,102,104,106,107]
Mercury	hypertrophy of seminiferous tubules with occlusions in the lumen vacuolated areas contained in the seminiferous tubules thinned tubules dilatated blood vessels the increased volume of the interstitium degeneration and disorganization of spermatogenic cells reduced number of spermatocytes down-regulation of Catsper 1 and 2 disruption of estrogen receptor [108,109,110,111,112,113,114]	reduced weight of epididymis decreased sperm count morphological aberrations elevated levels of ROS and lipid peroxidation disruption of estrogen receptor [110,114,115]	reduced sperm count higher incidence of immotile spermatozoa the damaged plasma membrane, axoneme elevated generation of ROS increased lipid peroxidation impaired fertilization ability altered DNA methylation decreased adenosine triphosphate inhibition of sperm creatine kinase [102,109,115,116,117,118,119]

## Data Availability

Not applicable.
